# Clinical evaluation of thrombotic microangiopathy: identification of patients with suspected atypical hemolytic uremic syndrome

**DOI:** 10.1186/s12959-016-0114-0

**Published:** 2016-10-04

**Authors:** Yu-Min Shen

**Affiliations:** Department of Internal Medicine, University of Texas Southwestern Medical Center, Dallas, TX 75390-8852 USA

**Keywords:** Thrombotic microangiopathy, Atypical hemolytic uremic syndrome, Thrombotic thrombocytopenic purpura, Complement dysregulation

## Abstract

Atypical hemolytic uremic syndrome (aHUS) is a rare genetic disorder caused by defective complement regulation resulting in thrombotic microangiopathy (TMA). Patients can present as children or adults. The syndrome consists of hemolytic anemia with schistocytosis, thrombocytopenia, significant renal damage, and/or other organ system dysfunction(s). Patients with aHUS may succumb to the complications of the disease with the very first manifestation; surviving patients often suffer from progressive organ dysfunction with significant morbidity and mortality despite plasma infusion or plasma exchange. Eculizumab, a humanized monoclonal antibody to C5, was approved for treatment of aHUS in 2011. This is an expensive but highly effective therapy changing the lives and improving the outcome of patients with aHUS. Making timely and accurate diagnosis of aHUS can be life-saving if eculizumab treatment is begun promptly. Finding a genetic mutation in a complement regulatory protein is diagnostic with the appropriate clinical syndrome, but at least 30 % of patients do not have defined or reported mutations. Thus the diagnosis rests on the clinical acumen of the physician. However, the clinical manifestations of aHUS are shared by other etiologies of thrombotic microangiopathy. While laboratory finding of undetectable ADAMTS13 activity defines TTP, distinguishing aHUS from the other causes of TMA remains an art. In addition, aHUS can be unmasked by conditions with enhanced complement activation, such as systemic lupus erythematosus, pregnancy, malignant hypertension, and hematopoietic stem cell transplantation. Thus if TMA occurs in the setting of enhanced complement activation, one must consider aHUS as an underlying etiology, especially if treatment of the condition does not resolve the TMA.

## Background

The clinical syndrome of organ dysfunction, microangiopathy hemolytic anemia and thrombocytopenia, most often caused by various forms of thrombotic microangiopathy, is a diagnostic enigma for the clinicians at the frontlines evaluating the critically ill. Historically, with poor understanding of pathophysiologic mechanisms, and plasma exchange being the only accepted therapy, recognition of the clinical syndrome was all that was needed to manage such patients. The precise understanding of the diagnostic entities within this syndrome in the last two decades, and availability of a specific therapeutic option, are forcing clinicians to retool their knowledge base in order to better serve their patients. This article reviews the distinction between atypical hemolytic uremic syndrome and other causes of thrombotic microangiopathy, especially thrombotic thrombocytopenic purpura, and proposes a diagnostic/management algorithm.

## Review

### What is thrombotic microangiopathy?

Thrombotic microangiopathy (TMA) is a pathologic condition with abnormalities in the blood vessel walls of arterioles and capillaries resulting in microvascular thrombosis [1]. There are several disease states that can lead to TMA (Table 1) [2, 3]. Clinically TMA is nearly always accompanied by microangiopathic hemolytic anemia (MAHA), a non-immune hemolytic anemia resulting from intravascular red cell fragmentation with schistocytosis and thrombocytopenia due to consumption. The direct antiglobulin test (DAT) is negative, and lactate dehydrogenase (LDH) is typically markedly elevated; bilirubin is modestly increased, while haptoglobin is undetectable. MAHA is most often caused by TMA, but intravascular devices such as prosthetic heart valve or left ventricular assist devices may also cause MAHA. In addition, many systemic disorders can be associated with MAHA with or without TMA (Table 2) [2, 3]. Rarely paroxysmal nocturnal hemoglobinuria and heparin-induced thrombocytopenia can present with MAHA and thrombocytopenia. It takes an astute clinician with the proper laboratory acumen to decipher the underlying cause of TMA/MAHA in a given patient.

**Table 1:** Causes of TMA

Thrombotic thrombocytopenic purpura (TTP)	Absence of ADAMTS13, the von Willebrand factor cleaving metalloprotease. Acquired due to autoimmune antibody to ADAMTS13, or hereditary (Upshaw-Schulman syndrome).
Infectious hemolytic uremic syndrome (ST-HUS)	Shiga toxins produced by Shigella dysenteriae and some serotypes of Escherichia coli (O157:H7 and O104:H4), cause direct damage to kidney epithelial and mesangial cells, and vascular endothelial cells. Rarely pneumococcus or other infectious agents with neuraminidase can expose the Thomsen-Friedenreich antigen on cell surfaces to result in hemolysis and direct endothelial injury.
Atypical or complement-mediated HUS	Hereditary deficiency of complement regulatory proteins (factor H, factor H related proteins, factor I, membrane cofactor protein, thrombomodulin) that normally regulate and restrict the activation of the alternative complement pathway, or hereditary abnormalities (factor B, C3) that accelerate the activation of the alternative complement pathway, leading to complement-mediated damage to vascular endothelium and kidneys. Acquired deficiency of complement factor H or factor I can be caused by autoimmune antibodies. Recessive mutations in diacylglycerol kinase epsilon (DGKE) is thought to result in a prothrombotic state with TMA in infancy (distinct from DGKE nephropathy). Plasminogen mutation was suggested to be the cause of aHUS in one case report.
Drug-induced TMA	Immune-mediated caused by drug-dependent antibodies that damage platelets, neutrophils and endothelial cells (quinine, gemcitabine, oxaliplatin and quetiapine). Dose-dependent toxicity-mediated caused by direct endothelial damage (gemcitabine, mitomycin, cyclosporine, tacrolimus, sirolimus, bevacizumab, oxymorphone).
Metabolism-mediated TMA	Disorders of intracellular vitamin B12 metabolism due to mutations in the MMACHC gene. Associated with elevated homocysteine and low methionine levels in plasma, with methylmalonic aciduria.

**Table 2:** Systemic disorders associated with TMA/MAHA (conditions with augmented or enhanced complement activation)

Pregnancy complications	
Severe hypertension	
Systemic infections (viremia, fungemia)	
Systemic malignancies (chemotherapy, tumor cell embolism)	
Systemic rheumatologic disorders (systemic lupus, scleroderma, catastrophic antiphospholipid syndrome)	
Hematopoietic stem cell transplantation (myeloablative drugs, immunosuppression, viremia/fungemia)	
Intravenous radiologic contrast media or exposures to biomaterials during vascular procedures	

### Hemolytic uremic syndromes and TTP

Hemolytic uremic syndrome (HUS) affects children and adults, and is characterized by MAHA, thrombocytopenia, and significant renal dysfunction. In most cases HUS is caused by Shiga-toxin bearing E coli; rarely pneumococcal infection can also lead to HUS [4]. However, in a small minority of patients, with so-called atypical hemolytic uremic syndrome (aHUS), no infectious agent is found. aHUS is a rare genetic disorder characterized by complement-mediated TMA resulting from mutations affecting the regulation of the alternative complement pathway. Numerous loss of function mutations in factor H, factor I, membrane cofactor protein, thrombomodulin, as well as gain of function mutations in C3 and factor B, have been discovered [5]. Patients present at all stages of life despite the genetic nature of the disease, and it is poorly understood what protects the patients from having disease manifestations until later in life. Historically, HUS and aHUS are discussed in the setting of thrombotic thrombocytopenic purpura (TTP), as the pathophysiology underlying these disorders were not known, and the clinical presentations are often indistinguishable. Since the reports of absolute ADAMTS13 deficiency (von Willebrand factor-cleaving metalloprotease) in TTP caused by autoimmune antibody to ADAMTS13 in 1998 by Tsai [6] and Furlan [7], TTP is now recognized as a distinct disorder with absence of detectable levels of ADAMTS13 activity as the defining feature of TTP [8]. Thus infectious HUS, atypical or complement-mediated HUS, and TTP have distinct pathophysiological mechanisms, and should no longer be regarded and treated as the same disease.

Previously, there was no utility in making a diagnostic distinction between HUS, aHUS and TTP as all patients were treated with plasma exchange. Plasma exchange remains the mainstay of therapy for TTP to remove the autoimmune antibody and to replenish the absent enzyme. Prognosis of TTP is excellent with early recognition of disease and prompt institution of plasma exchange [9]. Proper antibiotic therapy and supportive care is now considered the treatment of choice for infectious HUS. Since September 2011, eculizumab, a humanized monoclonal antibody against C5, was approved by the FDA for treatment of patients with TMA secondary to aHUS. With the high response rates of eculizumab and potential for renal recovery in aHUS patients [10], separating the rare patient with aHUS from infectious HUS and TTP is now a clinical urgency.

### Diagnostic approach to aHUS

When a critically ill patient with MAHA and thrombocytopenia is encountered, the differential diagnoses listed in Tables 1 and 2 should be considered [2, 3]. Several entities should be easily ruled out on clinical grounds, such as drugs, malignancy, pregnancy, hypertension and hematopoietic stem cell transplantation. Laboratory studies can identify rheumatologic disorders, B12 deficiency, antiphospholipid syndrome, and DIC. Therefore, the main differential consideration is TTP versus infectious HUS or atypical HUS. Infectious HUS can be identified with diarrheal illness with positive testing for the Shiga toxin; TTP now has a diagnostic test with ADAMTS13 activity [11–15]. However, testing with rapid turn-around time is not available at most institutions. Clinical findings and laboratory data may be helpful. TTP is not known to involve the lungs, and rarely results in liver dysfunction. Renal disease is typically mild in TTP patients (creatinine <1.7–2.3 mg/dL), while thrombocytopenia is more severe (<30 × 10^9^/L) compared to aHUS patients [16, 17], but overlap is considerable (unpublished data); the most recent patient diagnosed to have TTP at our institution had a creatinine of 5 mg/dL and platelet count of 47 × 10^9^/L. For atypical or complement-mediated HUS, while finding a pathologic mutation in the regulatory proteins of the alternative complement pathway is diagnostic, only a few reference laboratories offer the genetic analysis, and registry data show that at least 30 % of patients with aHUS do not have currently known mutations. Genetic testing is also expensive and time consuming; with the exception of Machaon Diagnostics, the results are not available for at least 3–4 weeks. Having a negative genetic analysis certainly does not rule out aHUS, and not all laboratories offer whole exome sequencing. Thus the diagnosis of aHUS remains clinical. The recommended approach is to initiate plasma exchange therapy while waiting for diagnostic tests (such as ADAMTS13 activity) [18]. If ADAMT13 activity is below the level of detection, TTP is diagnosed and plasma exchange is continued until resolution of the MAHA and thrombocytopenia. If ADAMTS13 is detectable, and there is no diarrheal illness, then atypical HUS is the most likely diagnosis; keep in mind that atypical HUS especially in adults may present with a diarrheal illness without Shiga-toxin, due to involvement of the gastrointestinal tract. Response to plasma exchange can be helpful, with TTP patients responding in 3–5 days, while the response in aHUS patients is at best partial with improvement of hematologic parameters while organ functions continue to worsen [18].

In summary, when a patient with MAHA, thrombocytopenia and organ dysfunction, a detectable level of ADAMTS13 activity and absence of Shiga-toxin induced diarrheal illness should raise the clinical suspicion for atypical or complement-mediated HUS. Empiric plasma exchange should be initiated while waiting for results of the ADAMTS13 activity and Shiga toxin testing. Less than optimal response in 5 days to empiric plasma exchange should heighten the suspicion that the patient does not have TTP and treatment for aHUS should be considered.

### Conditions with augmented or enhanced complement activation and aHUS

As mentioned in Table 2, there are several systemic conditions or disorders that can present with MAHA, thrombocytopenia, and organ dysfunction [2, 3]. These are in fact conditions with enhanced complement activation that can potentially unmask patients with mutations associated with aHUS. Registry data suggest that aHUS presents with conditions with enhanced complement activation nearly 70 % of the time [19]. When MAHA, thrombocytopenia, and organ dysfunction occur in patients with such a condition, care must be taken to monitor for prompt resolution of the hematologic abnormalities and organ dysfunction once the condition is resolved. Persistence of the hematologic abnormalities and/or organ dysfunction despite resolution of the condition with enhanced complement activation should lead to investigations for ADAMTS13 activity and/or Shiga toxin depending on the clinical situation, and consideration of empiric therapy with plasma exchange or initiation of eculizumab. It should be noted that whether the complement system is in fact enhanced or amplified is difficult to determine. Blood complement levels and detection of increased deposition of complement components in microvasculature remain investigational.

#### Pregnancy

Fakhouri et al. conducted a retrospective analysis of 100 adult female patients with aHUS. 21 had pregnancy-associated aHUS, and 79 % were post-partum. Mutations associated with aHUS were detected in 18 of the 21 patients. The risk for pregnancy associated aHUS was highest during a second pregnancy. Women with aHUS and documented genetic defects were more likely to have fetal loss and pre-eclampsia compared to those with aHUS but no genetic abnormalities [20].

Similar to non-pregnancy-associated aHUS, differentiating pregnancy-associated aHUS from other TMA is difficult due to clinical resemblance with other disorders including HELLP (hemolysis, elevated liver enzymes and low platelets), AFLP (acute fatty liver of pregnancy), and TTP. It is suggested that DIC picture and significant liver dysfunction would point towards HELLP or AFLP, and the absence of DIC and relatively normal liver enzymes are consistent with TTP or aHUS [21]. Pregnancy-associated TTP occurs in the late second or third trimester, with a median time at 23 weeks. This is thought to be related to the gradual rise of von Willebrand factor concentration with advancing gestation while ADAMTS13 activity decreases. In contrast, pregnancy-associated aHUS occurs mostly in the post-partum period, when effective control of the alternative complement pathway in the fluid phase is required [20].

**Fig. 1:**
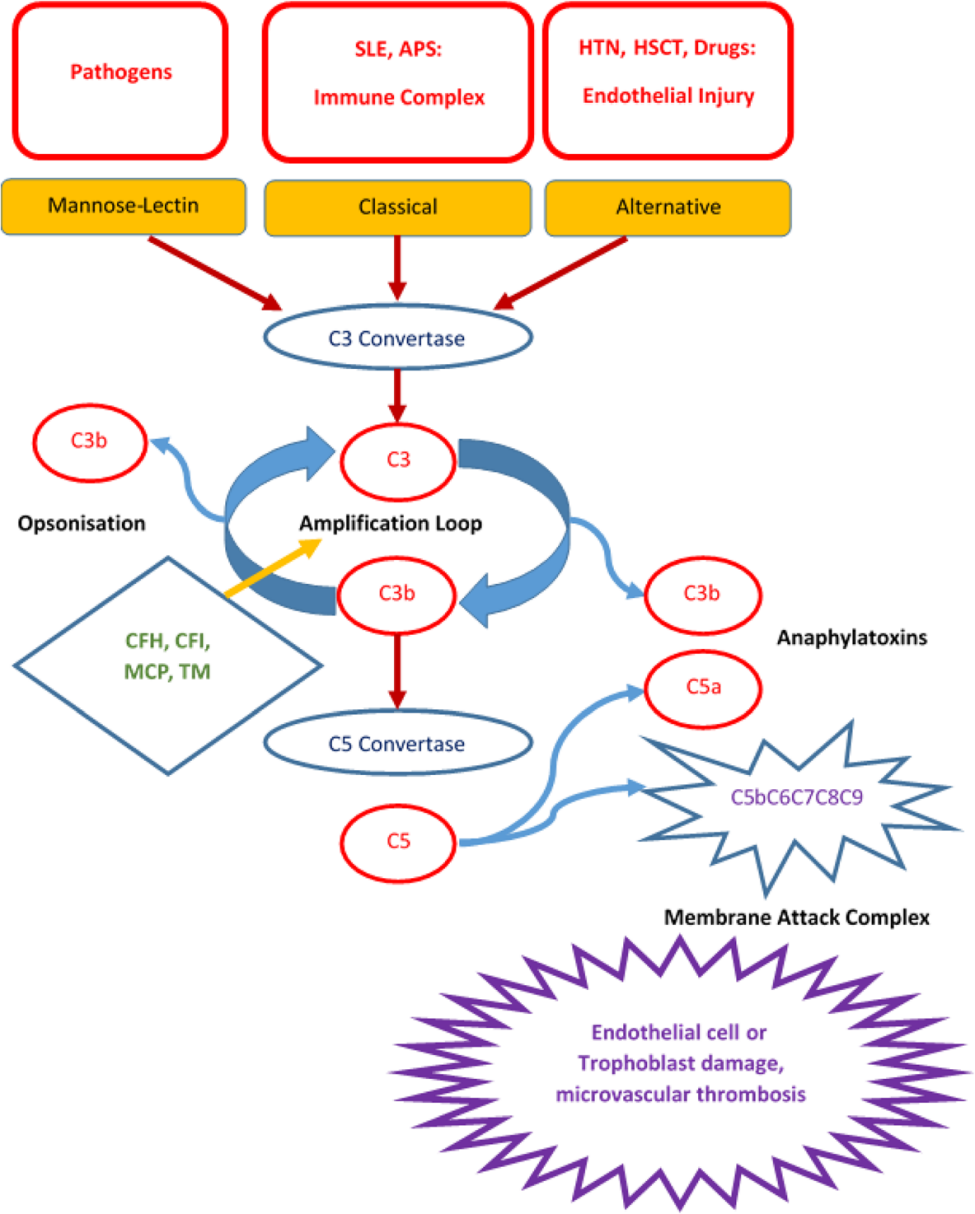
Complement system in the pathogenesis of thrombotic microangiopathies. SLE: systemic lupus erythematosus; APS: antiphospholipid syndrome; HTN: hypertension; HSCT: hematopoietic stem cell transplantation; CFH: complement factor H; CFI: complement factor I; MCP: membrane cofactor protein; TM: thrombomodulin

The PROMISSE study is a multi-institutional observation study designed to identify predictors of pregnancy outcome in patients with systemic lupus erythematosus or positive antiphospholipid antibodies. Patients were screened for mutations in membrane cofactor protein (MCP), complement factor I (CFI), and complement factor H (CFH). Forty out of 250 patients (17 %) enrolled had pre-eclampsia. Seven patients were found to have mutations, and all 7 were amongst the 40 who had pre-eclampsia. None of the 34 without pre-eclampsia screened had a mutation documented. Thus in this study 17.5 % of patients with history of pre-eclampsia in the setting of systemic lupus erythematosus or positive antiphospholipid antibodies had mutations associated with aHUS. It is likely that mutations associated with aHUS contributed to the development of pre-eclampsia in patients with lupus and/or antiphospholipid antibodies (Fig. 1) [22].

To illustrate these points, the first patient at our institution treated with eculizumab for aHUS presented during her third pregnancy at 20 weeks of gestation with marked hypertension, and edema. She was admitted to the obstetric service and treatment for presumed pre-eclampsia was begun. However, despite lowering her blood pressure to the near normal range and delivery of the nonviable fetus, her creatinine increased to 4 mg/dL, while her hemoglobin decreased to 6 g/dL and platelet count to 20 × 10^9^/L. She had modest amount of schistocytes on the blood smear, and ADAMTS13 activity was found to be normal on two separate occasions. She did not have diarrhea. She was empirically treated with 7 days of plasma exchange with minimal change in her clinical parameters. At that point presumptive diagnosis of pregnancy-associated aHUS was made, and she was initiated on eculizumab. By the second week of eculizuamb her hematologic abnormalities and serum creatinine began to normalize, with complete resolution of the disease manifestations by the fourth week. She has remained in remission as long as she is receiving eculizumab on schedule.

#### Malignant hypertension

While severe hypertension enhancing complement activity is not established, both infectious and atypical HUS, are often associated with significant hypertension. In contrast, TTP is not associated with malignant hypertension. This observed difference could be explained by a lower incidence and lesser degree of renal disease in TTP compared to HUS. Endothelial dysfunction is likely the common mechanism linking malignant hypertension and TMA. Endothelial dysfunction can result from either complement dysregulation, as seen in aHUS, or sheer stress in malignant hypertension [23]. Thus it is possible that endothelial injury from malignant hypertension can induce MAHA in a patient with underlying complement dysregulation (Fig. 1). An illustrative case is reported by Totina et al. A 10-month old child presenting with malignant hypertension and MAHA, but normal platelet count and normal renal function. Renal function and platelet count deteriorated during the hospital course, and renal biopsy demonstrated pathologic evidence of TMA. Molecular analysis showed a complement factor H mutation of unknown significance [24].

The distinction between malignant hypertension-associated TMA and TMA causing malignant hypertension is an important one as TMA causing malignant hypertension may well represent aHUS and respond to eculizumab. Absence of retinal changes (cotton-wool spots, flame hemorrhages, or retinal arteriolar narrowing) is consistent with TMA causing malignant hypertension, but not exclusively [23].

#### Systemic rheumatologic disorders

As mentioned above, complement dysregulation contributes to endothelial injury via the alternative pathway leading to TMA. In rheumatologic disorders such as lupus erythematosus and antiphospholipid syndrome, autoimmune antibodies form immune complexes, and activate the complement system via the classical pathway [25]. Active lupus is considered to be a risk factor for developing TMA in a Korean study [26]. Thus patients with genetic mutations associated with aHUS are particularly susceptible to TMA when there is tissue damage from uncontrolled rheumatologic disorders (Fig. 1). Specifically, for the integrity of the renal cells and glomerular basement membane, complement regulation is of particular importance [25]. Therefore, patients with rheumatologic disorders such as lupus erythematosus and genetic mutations associated with aHUS are particularly at risk for TMA.

Given the frequent occurrence of hemolytic anemia and thrombocytopenia in lupus patients, recognizing TMA in a lupus patient can be challenging. A Taiwanese retrospective analysis of lupus patients with TMA showed that while it is a rare occurrence at 1 % (consistent with other reports), a high mortality rate of 52 % is noted (prior to availability of eculizumab, despite plasma exchange in the majority of patients) [27]. TTP again is the main differential diagnosis when evaluating patients with TMA in the setting of lupus, occurring in 1–4 % of lupus patients with a high mortality [28]. Thus it is absolutely important to obtain ADAMTS13 testing in a lupus patient with TMA, and if ADAMTS13 activity is not absent, consider aHUS.

El-Husseini et al. reported a 24 year old female patient with lupus and lupus nephritis who developed MAHA, thrombocytopenia and worsening renal function despite intensification of treatment for lupus nephritis. Repeat kidney biopsy demonstrated both lupus nephritis and TMA. While genetic testing was not performed, patient’s clinical condition did not improve until eculizumab therapy was instituted, and thus patient is presumed to have developed aHUS in the setting of lupus and lupus nephritis [29]. This case again illustrates the importance of recognizing possible aHUS as a cause of persistent TMA when the underlying conditions with enhanced complement activation have been adequately treated. Investigations to identify risk features and determine the incidence of aHUS amongst lupus patients with TMA are vital.

#### Stem cell transplant

Transplant-associated TMA is a frequently encountered (~30 %) but poorly understood complication in recipients of hematopoietic stem cell transplantation (HSCT) [30]. It is noted to be more frequent in older patients and recipients of unrelated donor transplants, both of which are risk factors for acute graft versus host disease. It is more frequent in females, recipients of grafts with a major or bidirectional ABO blood group mismatch. The frequency of transplant-associated TMA correlated with GvHD severity [31]. With the current knowledge that endothelial cells are targets of acute GvHD (Fig. 1) [32], patients with genetic defects affecting complement regulation are thought to be more prone to develop transplant-associated TMA. Jodele et al. studied the genetic fingerprint of susceptibility for transplant-associated TMA, and found that HSCT recipients with multiple complement gene variants (≥3) are at high risk for severe transplant-associated TMA [33]. Thus transplant-associated TMA is likely a form of aHUS unmasked by the endothelial dysfunction resulting from the conditioning regimen, GvHD, and possibly the use of calcineurin inhibitors. In this particular type of TMA, absence of ADAMTS13 activity is generally not observed, and treatment with plasma exchange is rarely helpful [34]. Eculizumab not surprisingly is found to be effective in high risk transplant-associated TMA patients [30].

## Conclusion

**Fig. 2:**
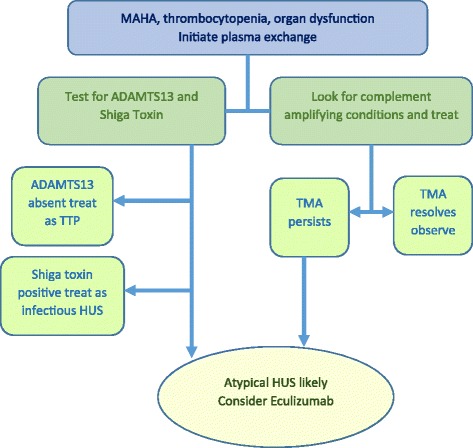
Diagnostic algorithm for patients with thrombotic microangiopathies (TMA). MAHA: microangiopathic hemolytic anemia; TTP: thrombotic thrombocytopenic purpura; HUS: hemolytic uremic syndrome

Atypical or complement-mediated HUS is a TMA caused by mutations affecting the regulation of alternative complement pathway. Clinical presentation with MAHA, thrombocytopenia, and significant organ dysfunction can mimic other TMA-causing entities. Absent ADAMTS13 activity rules in TTP, while diarrheal illness with Shiga-toxin producing bacteria points toward infectious HUS. When TMA occurs in the setting of conditions with enhanced complement activity such as pregnancy, rheumatic disorders, malignant hypertension, and HSCT, persistence or worsening TMA despite treatment of the associated conditions should raise the clinical suspicion for aHUS (Fig. 2). If aHUS is reasonably suspected, early intervention with anti-complement therapy should be considered especially when extra-renal complications are present; such patients are at increased risk of sudden death due to brain ischemia or cardiac complications.

## Abbreviations

ADAMTS13: A dysintegrin and metalloproteinase with thrombospondin type 1 motif, member 13
AFLP: Acute fatty liver of pregnancy
aHUS: Atypical hemolytic uremic syndrome
APS: Antiphospholipid syndrome
CFH: Complement factor H
CFI: Complement factor I
DAT: Direct antiglobulin test
DIC: Disseminated intravascular coagulation
GvHD: Graft versus host disease
HELLP: Hemolysis elevated liver enzymes and low platelets
HSCT: Hematopoietic stem cell transplantation
HTN: Hypertension
LDH: Lactate dehydrogenase
MAHA: Microangiopathic hemolytic anemia
MCP: Membrane cofactor protein
SLE: Systemic lupus erythematosus
TM: Thrombomodulin
TMA: Thrombotic microangiopathy
TTP: Thrombotic thrombocytopenic purpura

## References

[1] Tsai HM (2014). A mechanistic approach to the diagnosis and management of atypical hemolytic uremic syndrome. Transfus Med Rev.

[2] George JN, Charania RS (2013). Evaluation of patients with microangiopathic hemolytic anemia and thrombocytopenia. Semin Thromb Hemost.

[3] George JN, Nester CM (2014). Syndromes of thrombotic microangiopathy. N Engl J Med.

[4] Spinale JM, Ruebner RL, Kaplan BS, Copelovitch L (2013). Update on Streptococcus pneumoniae associated hemolytic uremic syndrome. Curr Opin Pediatr.

[5] Noris M, Remuzzi G (2009). Atypical hemolytic-uremic syndrome. N Engl J Med.

[6] Tsai HM, Lian EC (1998). Antibodies to von Willebrand factor-cleaving protease in acute thrombotic thrombocytopenic purpura. N Engl J Med.

[7] Furlan M, Robles R, Galbusera M, Remuzzi G, Kyrle PA, Brenner B (1998). von Willebrand factor-cleaving protease in thrombotic thrombocytopenic purpura and the hemolytic-uremic syndrome. N Engl J Med.

[8] Sarode R, Bandarenko N, Brecher ME, Kiss JE, Marques MB, Szczepiorkowski ZM (2014). Thrombotic thrombocytopenic purpura: 2012 American Society for Apheresis (ASFA) consensus conference on classification, diagnosis, management, and future research. J Clin Apher.

[9] George JN (2010). How I, treat patients with thrombotic thrombocytopenic purpura: 2010. Blood.

[10] Legendre CM, Licht C, Muus P, Greenbaum LA, Babu S, Bedrosian C (2013). Terminal complement inhibitor eculizumab in atypical hemolytic-uremic syndrome. N Engl J Med.

[11] Barbot J, Costa E, Guerra M, Barreirinho MS, Isvarlal P, Robles R (2001). Ten years of prophylactic treatment with fresh-frozen plasma in a child with chronic relapsing thrombotic thrombocytopenic purpura as a result of a congenital deficiency of von Willebrand factor-cleaving protease. Br J Haematol.

[12] Bianchi V, Robles R, Alberio L, Furlan M, Lammle B (2002). Von Willebrand factor-cleaving protease (ADAMTS13) in thrombocytopenic disorders: a severely deficient activity is specific for thrombotic thrombocytopenic purpura. Blood.

[13] Bitzan M, Schaefer F, Reymond D (2010). Treatment of typical (enteropathic) hemolytic uremic syndrome. Semin Thromb Hemost.

[14] Sadler JE (2008). Von Willebrand factor, ADAMTS13, and thrombotic thrombocytopenic purpura. Blood.

[15] Tsai HM (2010). Pathophysiology of thrombotic thrombocytopenic purpura. Int J Hematol.

[16] Coppo P, Schwarzinger M, Buffet M, Wynckel A, Clabault K, Presne C (2010). Predictive features of severe acquired ADAMTS13 deficiency in idiopathic thrombotic microangiopathies: the French TMA reference center experience. PLoS One.

[17] Zuber J, Fakhouri F, Roumenina LT, Loirat C, Fremeaux-Bacchi V, French Study Group for a HCG (2012). Use of eculizumab for atypical haemolytic uraemic syndrome and C3 glomerulopathies. Nat Rev Nephrol.

[18] Laurence J (2012). Atypical hemolytic uremic syndrome (aHUS): making the diagnosis. Clin Adv Hematol Oncol.

[19] Noris M, Caprioli J, Bresin E, Mossali C, Pianetti G, Gamba S (2010). Relative role of genetic complement abnormalities in sporadic and familial aHUS and their impact on clinical phenotype. Clin J Am Soc Nephrol.

[20] Fakhouri F, Roumenina L, Provot F, Sallee M, Caillard S, Couzi L (2010). Pregnancy-associated hemolytic uremic syndrome revisited in the era of complement gene mutations. J Am Soc Nephrol.

[21] Pourrat O, Coudroy R, Pierre F (2015). Differentiation between severe HELLP syndrome and thrombotic microangiopathy, thrombotic thrombocytopenic purpura and other imitators. Eur J Obstet Gynecol Reprod Biol.

[22] Salmon JE, Heuser C, Triebwasser M, Liszewski MK, Kavanagh D, Roumenina L (2011). Mutations in complement regulatory proteins predispose to preeclampsia: a genetic analysis of the PROMISSE cohort. PLoS Med.

[23] Mathew RO, Nayer A, Asif A (2016). The endothelium as the common denominator in malignant hypertension and thrombotic microangiopathy. J Am Soc Hypertens.

[24] Totina A, Iorember F, El-Dahr SS, Yosypiv IV (2013). Atypical hemolytic-uremic syndrome in a child presenting with malignant hypertension. Clin Pediatr (Phila).

[25] Java A, Atkinson J, Salmon J (2013). Defective complement inhibitory function predisposes to renal disease. Annu Rev Med.

[26] Kwok SK, Ju JH, Cho CS, Kim HY, Park SH (2009). Thrombotic thrombocytopenic purpura in systemic lupus erythematosus: risk factors and clinical outcome: a single centre study. Lupus.

[27] Chen MH, Chen MH, Chen WS, Mu-Hsin Chang P, Lee HT, Lin HY (2011). Thrombotic microangiopathy in systemic lupus erythematosus: a cohort study in North Taiwan. Rheumatology (Oxford).

[28] Vaidya S, Abul-ezz S, Lipsmeyer E (2001). Thrombotic thrombocytopenic purpura and systemic lupus erythematosus. Scand J Rheumatol.

[29] El-Husseini A, Hannan S, Awad A, Jennings S, Cornea V, Sawaya BP (2015). Thrombotic microangiopathy in systemic lupus erythematosus: efficacy of eculizumab. Am J Kidney Dis.

[30] Jodele S, Dandoy CE, Myers KC, El-Bietar J, Nelson A, Wallace G (2016). New approaches in the diagnosis, pathophysiology, and treatment of pediatric hematopoietic stem cell transplantation-associated thrombotic microangiopathy. Transfus Apher Sci.

[31] Martinez MT, Bucher C, Stussi G, Heim D, Buser A, Tsakiris DA (2005). Transplant-associated microangiopathy (TAM) in recipients of allogeneic hematopoietic stem cell transplants. Bone Marrow Transplant.

[32] Biedermann BC, Sahner S, Gregor M, Tsakiris DA, Jeanneret C, Pober JS (2002). Endothelial injury mediated by cytotoxic T lymphocytes and loss of microvessels in chronic graft versus host disease. Lancet.

[33] Jodele S, Zhang K, Zou F, Laskin B, Dandoy CE, Myers KC (2016). The genetic fingerprint of susceptibility for transplant-associated thrombotic microangiopathy. Blood.

[34] van der Plas RM, Schiphorst ME, Huizinga EG, Hene RJ, Verdonck LF, Sixma JJ (1999). von Willebrand factor proteolysis is deficient in classic, but not in bone marrow transplantation-associated, thrombotic thrombocytopenic purpura. Blood.

